# Electroacupuncture alleviated depression‐like behaviors in ventromedial prefrontal cortex of chronic unpredictable mild stress‐induced rats: Increasing synaptic transmission and phosphorylating dopamine transporter

**DOI:** 10.1111/cns.14200

**Published:** 2023-04-01

**Authors:** Xiaowen Cai, Mei Wu, Zhinan Zhang, Huacong Liu, Shengtao Huang, Jia Song, Siqiang Ren, Yong Huang

**Affiliations:** ^1^ School of Traditional Chinese Medicine Southern Medical University Guangzhou 510515 Guangdong China; ^2^ Guangdong‐Hong Kong‐Macao Greater Bay Area Center for Brain Science and Brain‐Inspired Intelligence, Key Laboratory of Mental Health of the Ministry of Education, Guangdong Province Key Laboratory of Psychiatric Disorders Southern Medical University Guangzhou 510515 Guangdong China

**Keywords:** depression, dopamine transporter, electroacupuncture, electrophysiology, trace amine‐associated receptor 1

## Abstract

**Aims:**

Electroacupuncture (EA) shows advantages in both clinical practice and depression animal models. Dopaminergic‐related dysfunction in the prefrontal cortex (PFC) may be a hidden antidepressant mechanism of EA, where dopamine transporter (DAT) plays an essential role. This study aimed to investigate the synaptic transmission and DAT‐related changes of EA in depression.

**Methods:**

Male Sprague–Dawley rats were subjected to 3‐week chronic unpredictable mild stress (CUMS). The successfully modeled rats were then randomly and equally assigned to CUMS, selective serotonin reuptake inhibitor (SSRI), and EA or SSRI + EA groups, followed by a 2‐week treatment respectively. After monitoring body weight and behavioral tests of all rats, the ventromedial PFC (vmPFC) tissue was collected for electrophysiology and the expression detection of DAT, phosphorylated DAT (p‐DAT), cyclic adenosine monophosphate (cAMP), protein kinase A (PKA), and trace amine‐associated receptor 1 (TAAR1).

**Results:**

Depressive‐like behaviors induced by CUMS were alleviated by EA, SSRI, and SSRI + EA treatments through behavioral tests. Compared with CUMS group, EA improved synaptic transmission in vmPFC by upregulating spontaneous excitatory postsynaptic currents amplitude. Molecularly, EA reversed the increased total DAT and p‐DAT expression as well as the decreased ratio of p‐DAT/total DAT along with the activation of TAAR1, cAMP, and PKA in vmPFC.

**Conclusion:**

We speculated that the antidepressant effect of EA was associated with enhanced synaptic transmission in vmPFC, and the upregulated phosphorylation of DAT relevant to TAAR1, cAMP, and PKA may be the potential mechanism.

## Graphical abstract

EA evoked synaptic transmission in vmPFC. EA upregulated phosphorylation of DAT relevant to TAAR1, cAMP, and PKA.

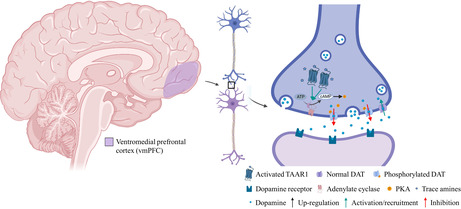

## 1 INTRODUCTION

Depression is a common mood disorder characterized by significant and persistent down in spirit which is not commensurate with the situation and is one of the leading disorders of disability.^1^ According to the World Health Organization, about 322 million people currently suffer from depression, causing a severe burden on society and families.^2^ In addition to low mood, patients with depression are often accompanied by other symptoms, such as cognitive impairment, slow thinking, and somatic symptoms. Therefore, the prevention and treatment of depression urgently need attention.

At present, antidepressant medication is the primary treatment for moderate–severe depression. However, the onset time of antidepressants is relatively long, requiring 6–8 weeks.^3^ Besides, long‐term use of these medications has certain adverse outcomes^4^ and relapse after withdrawal.^3^, ^5^ Complementary and alternative therapies, including acupuncture and moxibustion, can improve clinical symptoms while reducing side effects.^6^ Evidence‐based medicine and meta‐analysis have shown that acupuncture or electroacupuncture (EA) combined with antidepressants is more effective than drugs alone.^7^, ^8^ Acupuncture is a promising treatment for mild‐to‐severe depression, and EA can also alleviate concurrent symptoms, for instance, by improving the sleep quality of depressed patients.^9^ Animal studies also corroborated EA's effect on depression model rats,^10^, ^11^ but the mechanism of action is not precise yet.

Prefrontal cortex (PFC) is important in the pathogeny and treatment of depression^12^, ^13^ revealed by functional magnetic resonance imaging (fMRI) and positron emission tomography scanning on patients with depression who showed lower gray matter volume and cerebral blood flow value in the PFC,^14^, ^15^ while the cerebral blood flow in this area was closely associated with depressive symptoms.^16^ Recently, the ventral medial prefrontal cortex (vmPFC) has gained more and more attention in research about depression. The fMRI scanning on depressive patients showed decreased functional connectivity between vmPFC and other brain regions, correlated with increased anhedonia.^17^ Similar results also showed in chronic unpredictable mild stress (CUMS) depressive animal models that CUMS exposure induced microglial activation and inflammatory cytokine expression within this brain area.^18^ The vmPFC receives neural projection from many other regions and is involved in one of the four dopaminergic pathways (the mesocortical pathway, connecting the ventral tegmentum to the PFC). Given our previous isobaric tags for relative and absolute quantitation (iTRAQ) finding of dopaminergic changes in the PFC,^19^ the dopamine‐related mechanism in the vmPFC may be contributing to the alleviation of depressive symptoms.

Dopamine (DA) neurons are one of the crucial neurons involved in the onset of depression. Dopamine transporter (DAT) is one of the major factors that maintain the stability of DA in the synaptic cleft. Studies have shown that the availability of DAT is reduced in patients with depression.^20^ Growing evidence is proving that treatments aiming at DAT provided clinical improvement for patients with depression or typical behavioral retrieve for animal models,^21^, ^22^ enhancing DAT's role in the development of this affective disorder. Moreover, considering that selective serotonin reuptake inhibitors (SSRIs) line in the first‐line treatment for depression, clinical and fundamental researches have supported the participation of serotonin and dopamine systems in the downstream therapeutic mechanisms of SSRIs.^23^, ^24^ That is to say, these two systems have functional influences reciprocally, for example, DA signaling in SSRIs' antianxiety or antidepressant effect and SSRIs' effect on DAT.^23^, ^25^

DAT's on‐membrane stability is mostly regulated by post‐translational modifications, among which phosphorylation is the main factor. When DAT is phosphorylated, it can lead to reduced transport and elevated efflux of DA or enhanced transporter internalization.^26^ Protein kinase A (PKA) is one of the major kinases phosphorylating DAT, acting at Ser7 of the N‐terminal.^26^ Our former iTRAQ exploration of the mechanism of EA on CUMS rat model discovered DAT‐ and PKA‐related protein changes in PFC,^19^ which provided the possibility between DAT phosphorylation via PKA‐involved signal pathway and depression.

In recent years, trace amine‐associated receptor 1 (TAAR1) has attracted more and more attention in regulating monoamine neuron discharge and neurotransmitters in the central nervous system. TAAR1 is an intracellular G protein‐coupled receptor activated by trace amines, which trigger the accumulation of cyclic adenosine monophosphate (cAMP) via adenylyl cyclase (AC) activation and then increase the expression of PKA.^27^ In the early stage of this class, proteomics showed that the AC activating downstream of TAAR1 had significant changes after treatment. Combined with the changes in DAT, it suggested that TAAR1 could modulate the amount or function of DAT by activating downstream proteins.

The chromosome where TAAR1 is located has been consistently identified by linkage analyses as a susceptibility locus for schizophrenia and bipolar disorder,^28^ indicating the potential role of TAAR1 in the progress of depression. Evidence has proven the antidepressant effect of TAAR1 agonists on rodent and primate models.^29^, ^30^ What's more, a novel compound with agonist activity at TAAR1 could relieve negative symptoms (e.g., blunted affect and anhedonia) in mental disorders.^31^ Such evidence supports the antidepressant effect of TAAR1 activation, although the mechanism is still waiting to be clarified.

Therefore, to explore the relevant mechanism of EA on depression, we adopted a comprehensive plan of EA and antidepressant therapy to study the effect of this combination on DAT through the mediation of TAAR1. What's more, the electrophysiology changes in depression, among which synaptic plasticity is becoming an essential mechanism, have also been observed in patients and animal models. However, little research has learned the mechanism of EA in electrophysiology changes of depression. Hence, we applied patch clamp technology in vmPFC to verify the electrophysiology mechanism of EA in depression rats.

## 2 METHODS

### 2.1 Animals and grouping

One hundred male Sprague–Dawley rats were purchased from the Laboratory Animal Center of Southern Medical University (Guangdong, China; license No. SYXK [Yue] 2021‐0041), weighing 180–220 g at the beginning of the experiment. The rats were housed in a controlled environment, with ad libitum food and water. All experimental protocols were approved Southern Medical University Experimental Animal Ethics Committee (No. L2019046) and followed the guidelines of animal bioethics. Rats were divided into two groups at random: the control group (*n* = 20) and CUMS modeling group (*n* = 80). Except for control groups, other rats went through CUMS procedure for 21 consecutive days. The successful modeling rate reported was around 80%,^32^ and then, the depressed rats identified by behavioral analysis would be allocated randomly and equally to CUMS, SSRI, EA, and SSRI + EA groups (about *n* = 16 in each).

### 2.2 CUMS procedures

The CUMS procedure was performed as described previously^33^ with a slight modification. During CUMS model establishment, the rats were subjected to unpredictable stimuli including 3‐min tail‐clamping, eating, and drinking deprivation, 5‐min horizontal shaking without water ingestion, and 2‐h restraint. The above stress factors were adopted by the principle of randomness so that the rats cannot predict the occurrence of the stimulation. Rats received only one stimulus a day, and each stimulus was performed once every 4 or 5 days on average.

### 2.3 Intervention

All rats received a 2‐week (1 time/day) intervention. Rats in the EA and SSRI + EA group received EA stimulation on GV20 (at the midpoint between the auricular apices) and GV29 (at the midpoint between the medial ends of the two eyebrows).^34^, ^35^ Disposable acupuncture needles (0.16 mm × 13 mm; Hwato Appliance Factory) were inserted into both acupoints horizontally to 5 mm depth toward the tip of nose. Following the insertions, the needles were connected to electrodes for electrical stimulation with continuous waves (2 Hz in frequency, and 1 mA in intensity). The stimulus intensity was preferred when the rat's head trembled slightly. The whole EA treatment was implemented for 30 min each time and once per day, during which the rats were kept awake and calm in single cages in case of mutual disturbance. 0.9% saline and paroxetine were given to EA and SSRI + EA groups respectively after EA treatment.

The rats in the SSRI group received paroxetine (1.8 mg/kg) as described in previous studies^36^, ^37^ on a daily level after 30‐min flexible fixation (covered the rat with a towel for 1 min and then softly wound a piece of paper self‐adhesive tape around its neck) the same as the EA group. The administration of the drug was performed orally by gastric gavage. The same dosage of saline (per kg as paroxetine) was also applied to the EA group. The control group and the CUMS group also needed gastric gavage and fixation mentioned above to eliminate experimental errors and interference.

### 2.4 Behavioral tests

Weighing and various behavioral tests such as the open field test (OFT) and sucrose preference test (SPT) were done as described previously^38^ on all rats before and after the process of CUMS, and weekly during 2‐weeks treatment. These results were used to investigate the degree of anhedonia and behavioral despair altered by modeling and intervention. All behavioral tests were done by the investigator who was unaware of the treatment of each animal.

#### 2.4.1 Open field test

During each test, rats were individually and gently placed in the center of a black open field arena measuring 100 × 100 × 50 cm (*L* × *W* × *H*) and allowed to freely explore for 5 min. A camera was mounted above the open box for recording locomotor activity. Locomotion was determined by measuring the total distance traveled in the open field and the time spent in the center of the arena. The testing apparatus was thoroughly cleaned following each test using 70% ethanol.

#### 2.4.2 Sucrose preference test

All rats underwent 72‐h adaptive training before the start of SPT. Each rat was housed in an individual cage. During the first 24 h, rats received two bottles of 1% sucrose solution, and in the second 24 h, one bottle of sucrose solution was changed to pure water. After that, all bottles and food were deprived in the third 24 h. After adaption, each rat got two bottles of 1% sucrose solution and pure water (100 mL each) as well as food for them to access freely for 1 h. Afterward, two bottles were weighed and sucrose preference was defined as follows: sucrose preference percentage (%) = sucrose solution consumption (g)/(sucrose solution consumption [g] + water consumption [g]) × 100%.

### 2.5 Electrophysiology

Whole‐cell recordings were performed on acute brain slices as previously reported.^39^ Twelve rats randomly chosen from control, CUMS, and EA groups (*n* = 4 in each group) were deeply anesthetized with isoflurane gas and decapitated. The rat brains were removed rapidly and stuck to the slicing machine base before being placed in an artificial cerebrospinal fluid (ACSF) solution at 0°C. The ACSF for cutting consisted of (in mM) 2 KCl, 1.3 NaH_2_PO_4_⋅2H_2_O, 12 MgSO_4_, 0.2 CaCl_2_⋅2H_2_O, 26 NaHCO_3_, 10 d‐Glucose, and 220 sucrose. The brains were then sectioned into 300 μm coronal slices containing vmPFC with a vibratome (Leica vt1000s). After that, these brain slices were transferred into an incubator filled with ACSF for recording consisting of (in mM) 126 NaCl, 3 KCl, 1.25 NaH_2_PO_4_⋅2H_2_O, 1 MgSO_4_, 2 CaCl_2_⋅2H_2_O, 26 NaHCO_3,_ and 10 d‐Glucose for 30‐min incubation at 34°C, followed by a 2‐h incubation at room temperature. During cutting and incubation, the ACSF solution was continuously aerated with 5% carbon dioxide and 95% oxygen. The pH of all solutions was adjusted to 7.2 and the osmotic pressure was 280–300 mOsm/L.^40^

vmPFC neurons were viewed under upright fixed microscope (Nikon; Eclipse FN1) and recorded with an amplifier (HEKA; EPC‐10). Current data were collected with (HEKA; Patchmaster). To explore synaptic transmission, the frequency, and amplitude of spontaneous excitatory and inhibitory postsynaptic currents (spEPSCs and spIPSCs) were recorded using a voltage‐clamping mode. spEPSCs were recorded with the membrane potential clamped at −70 mV. spIPSCs were recorded with the membrane potential clamped at 0 mV. spEPSCs and spIPSCs were recorded from each cell for 5 min,^41^ followed by offline analysis using MiniAnalysis software (v6.0.7; Synaptosoft).

### 2.6 Immunohistochemistry

The left vmPFC of random 44 rats (*n* = 8 in CON, CUMS, and EA groups; *n* = 10 in SSRI and SSRI + EA groups) were separated and immersed in 4% paraformaldehyde for 24 h before being embedded in paraffin and sectioned. Before immunostaining, 4‐μm‐thick brain tissue sections were dewaxed in xylene, rehydrated through decreasing concentrations of ethanol, and washed in phosphate‐buffered saline (PBS). The immunohistochemistry (IHC) procedure followed the protocol of IHC SP‐kit (Rabbit; Bioss). Briefly, after PBS washing, the samples were heated by microwave in PBS for antigen retrieval and immersed with 3% H_2_O_2_ at room temperature for 15 min to block endogenous horseradish peroxidase activity. Afterward, each section was incubated with normal goat serum for 15 min and then incubated with primary antibody (1:1500; anti‐DAT antibody, Proteintech; Catalog number: 22524‐1‐AP) at 4°C overnight. On the second day, the slides were washed in PBS and incubated with a corresponding second antibody in the kit at room temperature for 30 min and stained with diaminobenzidine (keygen biotech). After staining, sections were dehydrated through increasing concentrations of ethanol and xylene, followed by mounting with neutral balsam. Images of brain tissue slices were captured with microscopic observation (Olympus) with 200× magnification and the IHC scores were calculated by the software ImageJ (version 1.4.3.67; National Institutes of Health, Bethesda, MD, USA), which showed the percent contribution of different grades of positive.

### 2.7 Western blot

The vmPFC of random 24 rats (*n* = 4 in CON, CUMS, and EA groups; *n* = 6 in SSRI and SSRI + EA groups) were lysed in RIPA buffer (50 mM Tris–HCl pH 8, 150 mM NaCl, 1% sodium deoxycholate, 1% NP‐40, 1 mM dithiothreitol, and 0.1% sodium dodecyl sulphate (SDS) for cultures or 1% SDS for tissue) containing protease and phosphatase inhibitors (Beyotime Institute of Biotechnology, Shanghai, China). The lysis process was continued for 30 min at 4°C and then centrifuged at 12,000 rpm at 4°C for 15 min to obtain the supernatant, with which the protein concentration was established by a Bicinchoninic Acid Protein Assay Kit (Beyotime Institute of Biotechnology). After that, total tissue lysates were denatured in sodium dodecyl sulphate‐polyacrylamide gel electrophoresis (SDS‐PAGE) sample loading buffer (League) followed by heating at 95° for 5 min. Before electrophoresis, equal quantities of sample protein (30 μg) were separated on 12% SDS‐PAGE gels. When it was finished, the SDS‐PAGE gels were transferred onto polyvinylidene fluoride (PVDF) membranes (Millipore). After blocking with a 5% bovine serum albumin solution in Tris‐buffered salinewith 0.05% Tween‐20 for 2 h at room temperature, membranes were incubated overnight at 4°C with the following primary antibodies: anti‐DAT (1:1000; Proteintech; Catalog number: 22524‐1‐AP), anti‐DAT‐phospho (T53) (1:1000; Abcam; Catalog number: ab183486), anti‐TAAR1 (1:1000; Thermo Fisher Scientific; Catalog number: PA5‐95704), anti‐PKA (1:20,000; Abcam; Catalog number: ab76238), and anti‐GAPDH (1:2000, Proteintech; Catalog number: HRP‐60004). On the next day, the PVDF membranes were washed and incubated with HRP‐conjugated Affinipure Goat Anti‐Rabbit IgG(H + L) (Proteintech; 1:2000; Catalog number: SA00001‐2) for 1 h at room temperature. Finally, immunoreactivity was visualized using enhanced chemiluminescence (Millipore) with darkroom development techniques for chemiluminescence (Proteinsimple; Fluorchem E) and the gray intensity of the protein bands was quantified by densitometric analysis (ImageJ).

### 2.8 Enzyme‐linked immunosorbent assay

The right vmPFC (*n* = 8 in CON, CUMS, and EA groups; *n* = 10 in SSRI and SSRI + EA groups) was rinsed with PBS, homogenized in cold 0.1 N HCl at a 1:5 ratio (w/v), and centrifuged (10,000 *g* for 15 min, at 4°C) for supernatant collection. After neutralized with 1 N NaOH, the cAMP content in the supernatant was measured using the mouse cAMP ELISA kit (R&D System China Co., Ltd.) according to the manufacturer's protocol. Protein levels of cAMP in vmPFC were normalized to the total protein levels of each supernatant respectively. Finally, absorbance was measured at 450 nm with a microplate reader (Thermo Scientific; Multiskan FC).

### 2.9 Statistical analysis

All data in the present study were expressed as the mean ± standard error of the mean and analyzed using SPSS 22.0. Shapiro–Wilk test was used to evaluate the distribution of the data. The normally distributed data were first analyzed with one‐way analysis of variance followed by least significance difference post hoc test as multiple comparisons between groups if satisfying homogeneity of variance, while Brown–Forsythe test followed by Dunnett's T3 post hoc test was used on the contrary. Data that did not exhibit a normal distribution were analyzed via a non‐parametric equivalent. A value of *p* < 0.05 was considered statistically significant for analysis. The graphs and line charts were finished by GraphPad Prism 8.0 (GraphPad Software, Inc.).

## 3 RESULTS

### 3.1 EA could reverse the depression‐like behaviors of CUMS rats

**FIGURE 1:**
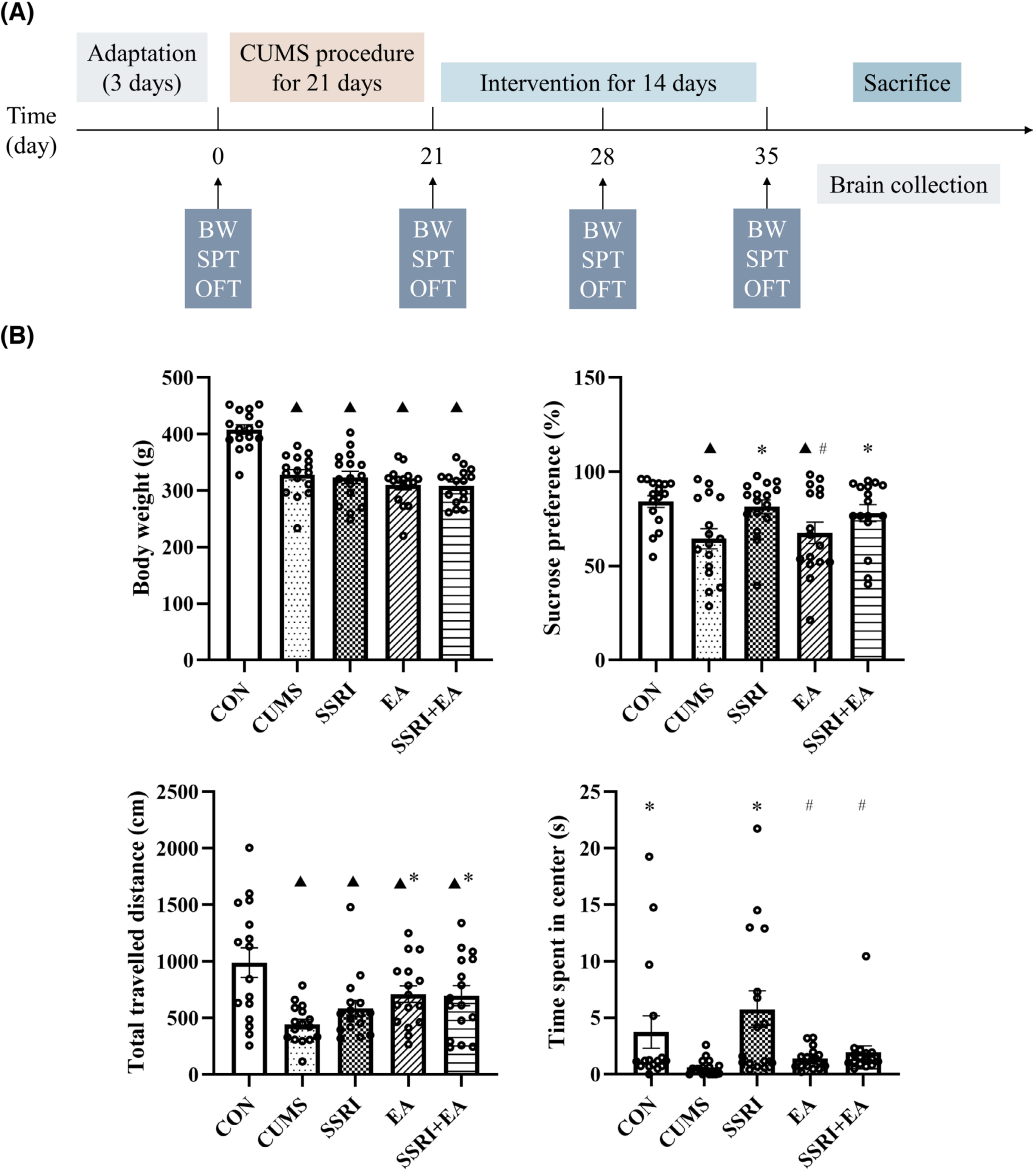
Time axis of experimental procedures and changes in body weight and behavioral analysis among groups. (A) Schedule of CUMS modeling, behavioral tests, intervention, and sample collection. CUMS, chronic unpredictable mild stress; BW, body weight; OFT, open field test; SPT, sucrose preference test. (B) Data were presented as mean ± standard error of the mean (*n* = 16). ^▲^ *p* < 0.05 compared with CON, *p < 0.05 compared with CUMS, and ^#^ *p* < 0.05 compared with SSRI, by one‐way analysis of variance with least significance difference post hoc comparison (Body weight: *F* = 20.957, *p* < 0.05. Sucrose preference: *F* = 3.588, *p* = 0.010. Total traveled distance: *F* = 5.442, *p* = 0.001. Time spent in center: *F* = 4.191, *p* = 0.004. All df = 4). EA, electroacupuncture; SSRI, selective serotonin reuptake inhibitor.

Behavioral analyses including SPT and OFT were used to evaluate the depression‐like behavior of rats, and the body weights of all rats were measured as well. The results of these behavioral analyses were non‐significantly different before modeling. The CUMS model was successfully established and maintained till the end of the intervention (seen in CUMS group), supported by the significantly decreased body weight, sucrose preference, total traveled distance, and time spent in the center in Figure 1. The increased sucrose preference of SSRI and SSRI + EA groups proved the amelioration of combined therapy of SSRI and EA on CUMS rats, although the body weight of rats among CUMS, SSRI, EA, and SSRI + EA groups was insignificantly different. The OFT was conducted to measure the effects on locomotion activity of rats exposed to CUMS. EA and SSRI + EA significantly increased the total traveled distances compared with CUMS group, while SSRI promoted the time spent at the center of the open field (Figure 1). The results above suggested that EA and SSRI + EA could improve the depression‐like behaviors of rats like SSRI.

### 3.2 EA improved the declined excitatory synaptic transmission caused by CUMS in vmPFC

**FIGURE 2:**
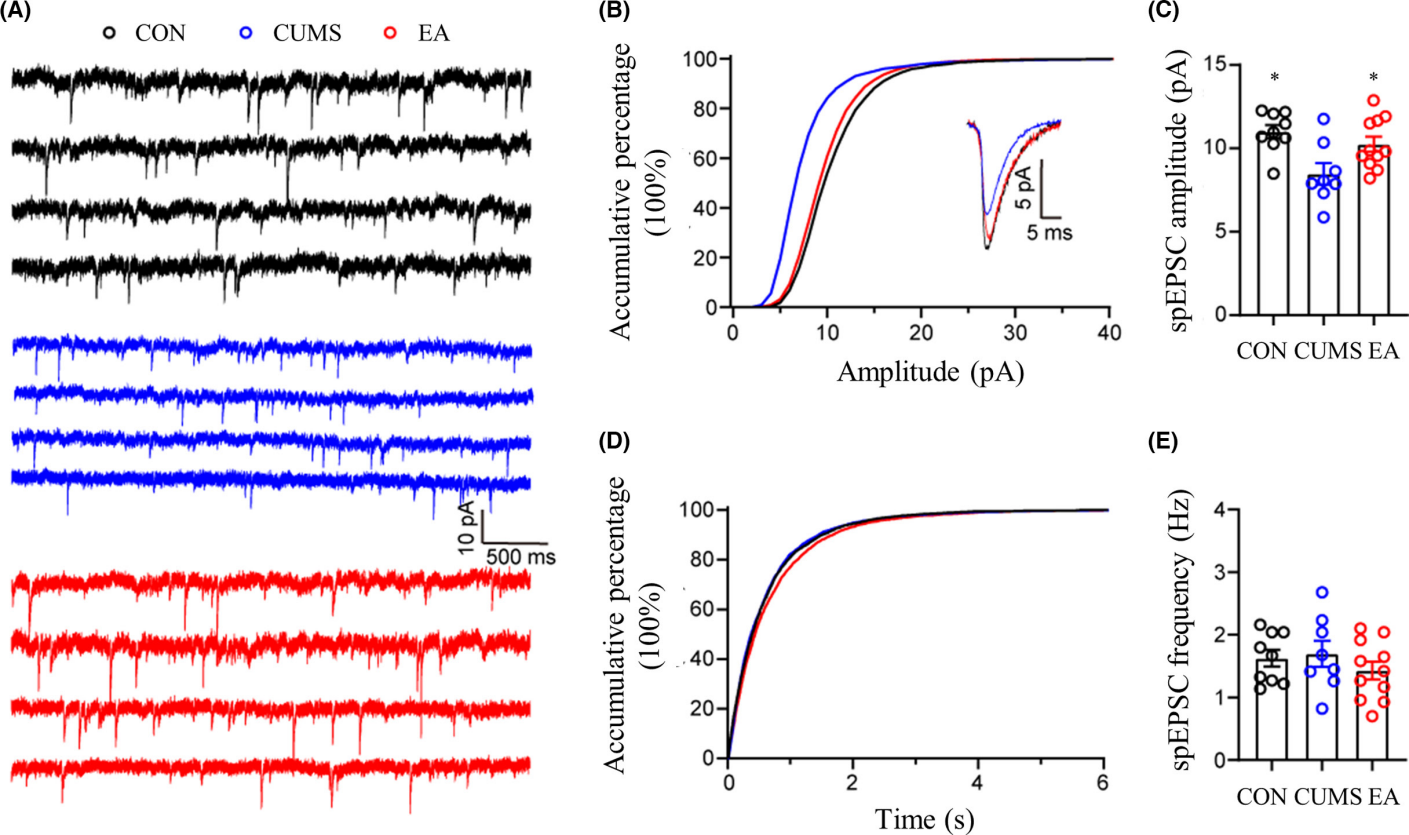
Amplitude and frequency changes of spEPSC among CON, CUMS, and EA groups. (A) Representative samples of spEPSC from each group. (B, C) The accumulative percentage and comparison of spEPSC amplitude among CON, CUMS, and EA groups. (D, E) The accumulative percentage of spEPSC inter‐event interval and comparison of spEPSC frequency among CON, CUMS, and EA groups. Data were presented as mean ± standard error of the mean (*n* = 4). *p < 0.05 compared with CUMS by one‐way analysis of variance with least significance difference post hoc comparison (spEPSC amplitude: *F* = 6.216, *p* = 0.006. spEPSC frequency: *F* = 0.786, *p* = 0.467. All df = 2). CUMS, chronic unpredictable mild stress; EA, electroacupuncture; spEPSC, spontaneous excitatory postsynaptic current.

**FIGURE 3:**
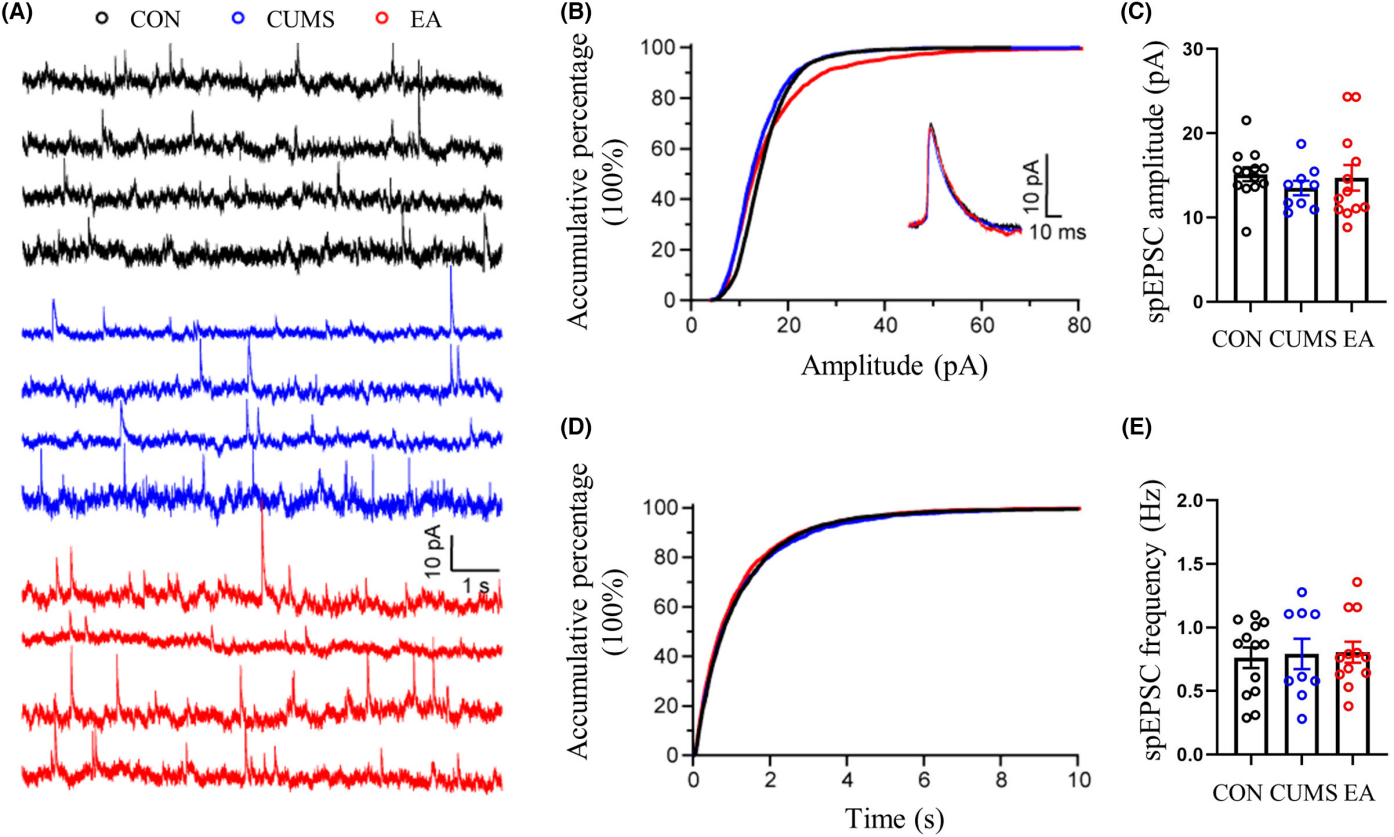
Amplitude and frequency changes of spIPSC among CON, CUMS, and EA groups. (A) Representative samples of spIPSC from each group. (B, C) The accumulative percentage and comparison of spIPSC amplitude among CON, CUMS, and EA groups. (D, E) The accumulative percentage of spIPSC inter‐event interval and comparison of spIPSC frequency among CON, CUMS, and EA groups. Data were presented as mean ± standard error of the mean (*n* = 4). No significant difference was detected among the amplitude and frequency comparison of CON, CUMS, and EA groups (spIPSC amplitude: *F* = 0.495, *p* = 0.615. spIPSC frequency: *F* = 0.064, *p* = 0.939. All df = 2). CUMS, chronic unpredictable mild stress; EA, electroacupuncture; spIPSC, spontaneous inhibitory postsynaptic current.

Previous studies suggest that depression‐like behaviors are related to a perturbed synaptic transmission in vmPFC,^42^, ^43^ so we hypothesized that EA treatment may ameliorate the depression‐like phenotype by improving the perturbed synaptic transmission in vmPFC. To prove this hypothesis, whole‐cell recordings were performed on excitatory pyramidal neurons in vmPFC. spEPSC and spIPSC were recorded with membrane potential maintained at −70 and 0 mV respectively (Figures 2 and 3). Our results showed that the amplitude of spEPSC but not spIPSC was significantly reduced in CUMS modeling mice, whereas the frequency of both spEPSC and spIPSC was not influenced (Figure 2B,C). These data suggest that the declined excitatory synaptic transmission is pertinent to the pathogenesis of depression, consistent with previous reports.^43^, ^44^ EA was then applied to CUMS modeling mice. As expected, EA treatment reversed the reduced amplitude of spEPSC. At the same time, EA had no significant influence on spEPSC frequency as well as spIPSC amplitude and frequency (Figures 2D,E and 3), indicating EA could specifically improve the declined excitatory synaptic transmission caused by CUMS. Taken together, our results suggest that CUMS induces the pathogenesis of depression by suppressing the excitatory synaptic transmission in vmPFC, and EA ameliorates the depression‐like phenotypes most likely by reversing the declined excitatory synaptic transmission in vmPFC. DA‐induced synaptic plasticity was associated with DAT^45^ and proven to act in depression with mPFC involved.^46^ Therefore, we probed into the DAT‐related changes for further studying the relationship between the DA system and depressive‐like behaviors.

### 3.3 EA reversed the increased DAT expression and enhanced phosphorylation in vmPFC after CUMS

**FIGURE 4:**
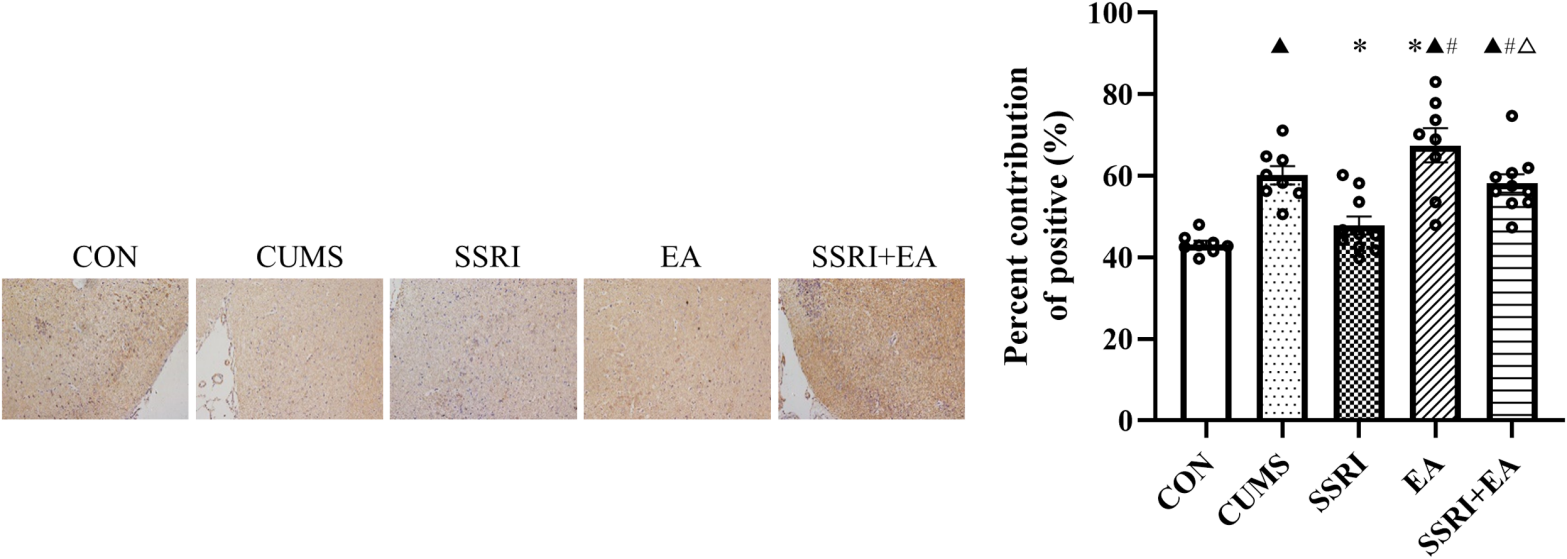
Expression of DAT by immunohistochemistry. Data were presented as percent contribution of positive (%) (*n* = 8 in CON, CUMS, and EA groups; *n* = 10 in SSRI and SSRI + EA groups). ^▲^ *p* < 0.05 compared with CON, *p < 0.05 compared with CUMS, ^#^ *p* < 0.05 compared with SSRI, and ^△^ *p* < 0.05 compared with EA by one‐way analysis of variance with least significance difference post hoc comparison (*F* = 0.064, df = 4, *p* < 0.05). CUMS, chronic unpredictable mild stress; DAT, dopamine transporter; EA, electroacupuncture; SSRI, selective serotonin reuptake inhibitor.

**FIGURE 5:**
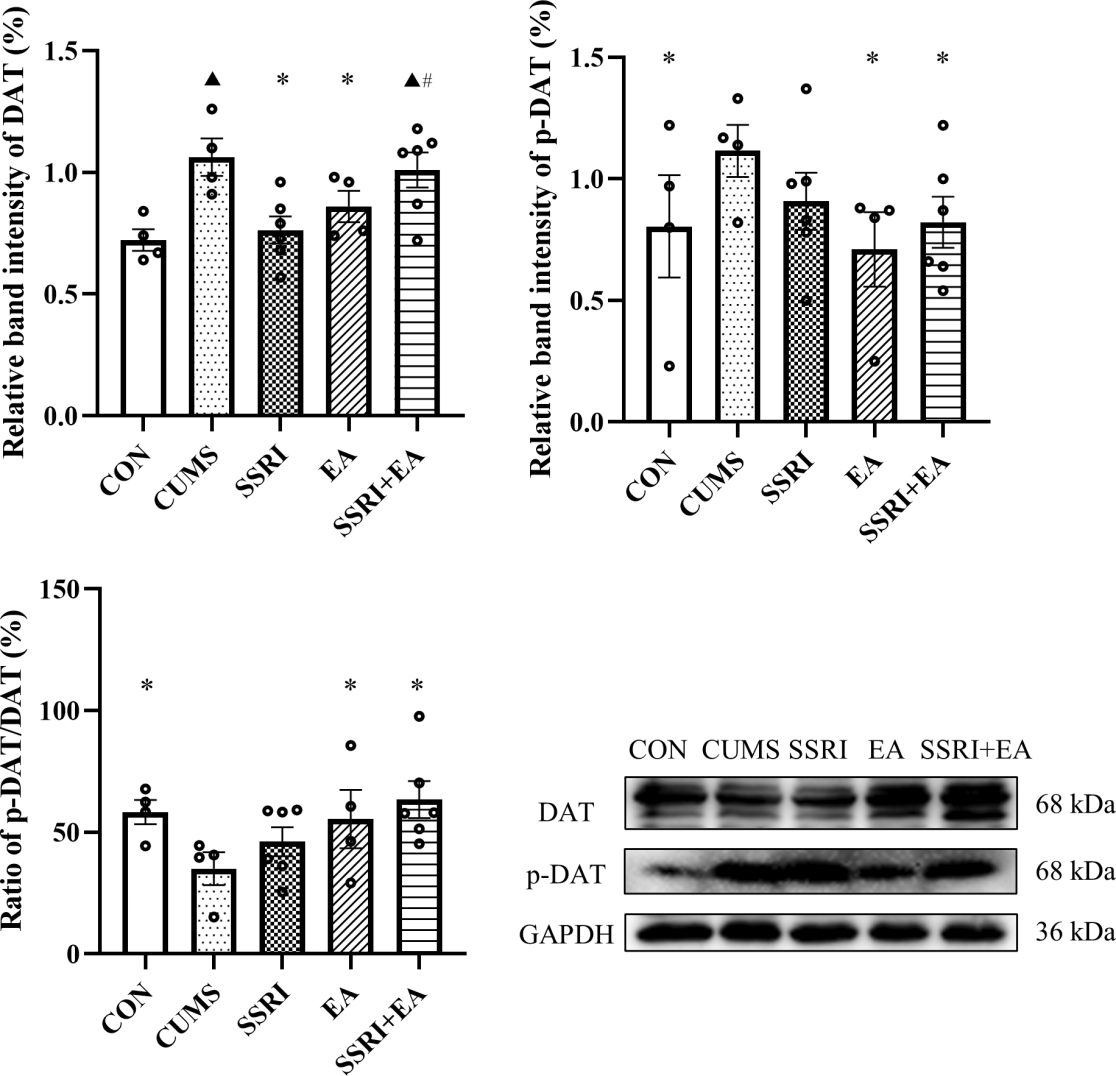
Expression of total DAT and p‐DAT by Western blot and the ratio of p‐DAT/DAT. Data were presented as mean ± standard error of the mean (*n* = 4 in CON, CUMS, and EA groups; *n* = 6 in SSRI and SSRI + EA groups). ^▲^ *p* < 0.05 compared with CON, *p < 0.05 compared with CUMS, and ^#^ *p* < 0.05 compared with SSRI by one‐way analysis of variance with least significance difference post hoc comparison (Relative band intensity of DAT: *F* = 5.059, *p* = 0.006. Relative band intensity of p‐DAT: *F* = 2.911, *p* = 0.032. All df = 4). CUMS, chronic unpredictable mild stress; DAT, dopamine transporter; EA, electroacupuncture; p‐DAT, phosphorylated DAT; SSRI, selective serotonin reuptake inhibitor.

The expression of DAT increased after CUMS modeling, supported by a larger positive area by IHC and relative band intensity by western blot (Figures 4 and 5). Besides, the expression of phosphorylated DAT (p‐DAT) in vmPFC also raised in CUMS group, which was similar to the changes in DAT, but the ratio of p‐DAT/total DAT detected by Western blot decreased after CUMS modeling (Figure 5). After treatments, EA reversed the excessive DAT and deficient ratio of p‐DAT/total DAT, while SSRI only took effect on regulating DAT expression and SSRI + EA just increased the ratio (Figure 5). Besides, the SSRI group presented a smaller positive area and lower expression of DAT than the SSRI + EA group by IHC and Western blot respectively, but EA increased the positive expression of DAT than both SSRI and SSRI + EA groups by IHC (Figures 4 and 5). In addition, the level of p‐DAT and the ratio of p‐DAT/total DAT showed non‐significantly differences in the comparison of these three groups (Figure 5). The results indicated that the CUMS‐caused depression‐like behavior was related to DAT phosphorylation in vmPFC, which was also a potential mechanism of EA in ameliorating depression in rats.

### 3.4 EA activated TAAR1, cAMP, and PKA along with the phosphorylation of DAT

**FIGURE 6:**
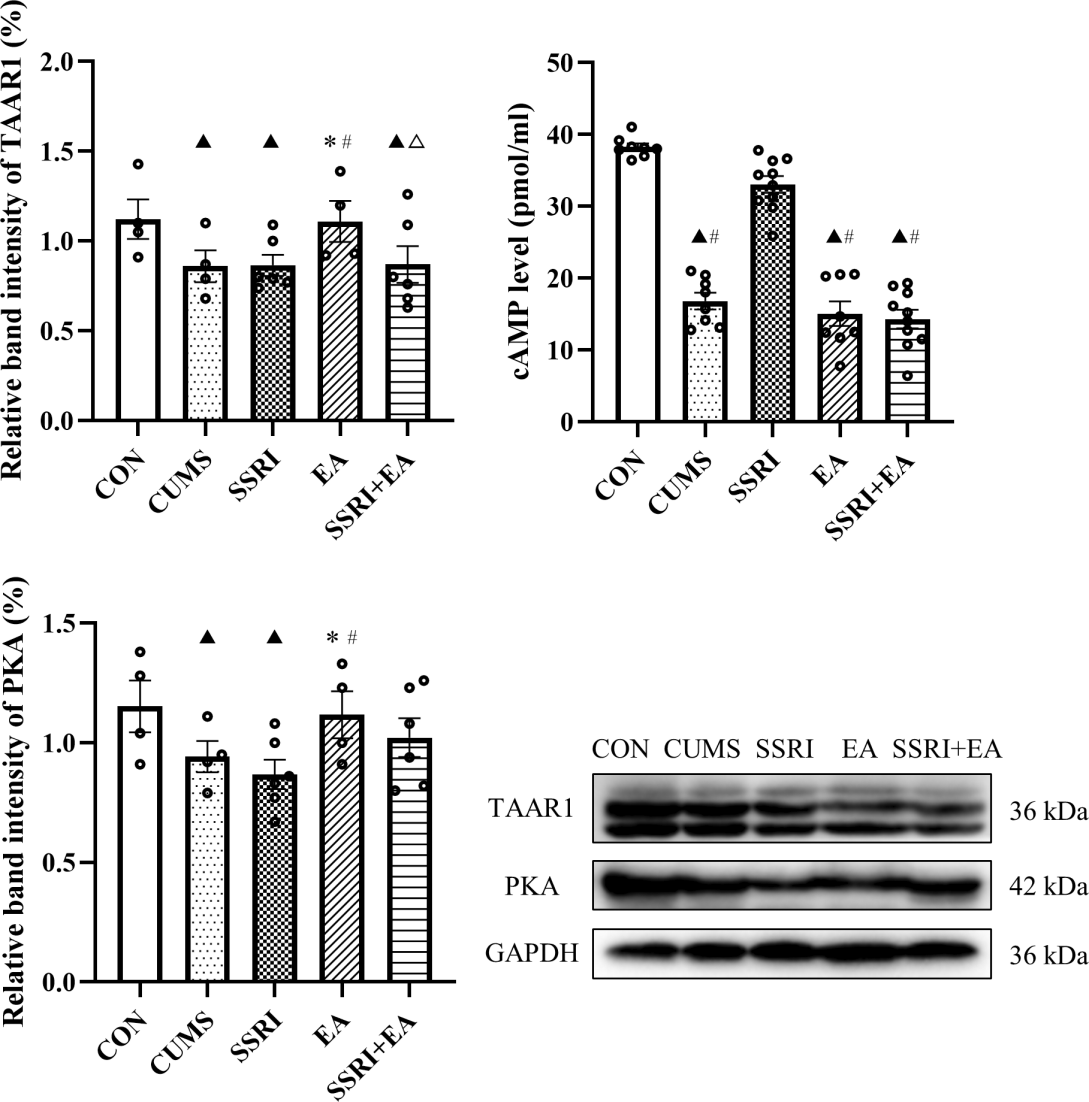
Changes in TARR1, cAMP, and PKA among groups. Data were presented as mean ± standard error of the mean (vmPFC for enzyme‐linked immunosorbent assay: *n* = 8 in CON, CUMS, and EA groups; *n* = 10 in SSRI and SSRI + EA groups. vmPFC for western blot: *n* = 4 in CON, CUMS, and EA groups; *n* = 6 in SSRI and SSRI + EA groups). ^▲^ *p* < 0.05 compared with CON, *p < 0.05 compared with CUMS, ^#^ *p* < 0.05 compared with SSRI, and ^△^ *p* < 0.05 compared with EA by one‐way analysis of variance with least significance difference post hoc comparison (Relative band intensity of TAAR1: *F* = 3.310, *p* = 0.023. CAMP level: *F* = 82.490, *p* < 0.05. Relative band intensity of PKA: *F* = 3.955, *p* = 0.009. All df = 4). CUMS, chronic unpredictable mild stress; cAMP, cyclic adenosine monophosphate; EA, electroacupuncture; PKA, protein kinase A; SSRI, selective serotonin reuptake inhibitor; TAAR1, trace amine‐associated receptor 1; vmPFC, ventromedial prefrontal cortex.

The expression of cAMP (detected by enzyme‐linked immunosorbent assay), PKA, and TAAR1 (detected by Western blot) all decreased after CUMS modeling (Figure 6). Compared with CUMS group, SSRI only reversed the cAMP level and did not influence PKA or TAAR1, while interestingly, EA demonstrated significant activation on PKA and TAAR1 but not cAMP compared with CUMS group (Figure 6). In the comparison of the three intervention groups, the SSRI group presented a higher cAMP level than the EA group, but the changes in PKA and TAAR1 were opposite (Figure 6). Additionally, the cAMP level in the SSRI group and TAAR1 level in the EA group were higher than those of the SSRI + EA group (Figure 6). PKA is dependent on cAMP and is known as one of the main phosphorylases acting on DAT. Although cAMP and PKA did not coincide with each other in EA group, the increased PKA indicated the activation of the cAMP/PKA signaling to a certain degree by EA, and such activation was associated with TAAR1. Taking together, the phosphorylation of DAT may be the downstream effect of the activated TAAR1, cAMP, and PKA.

## 4 DISCUSSION

CUMS is a well‐developed depression model proven by plenty of research. From the decreased body weight, sucrose preference, total distance traveled, and time in the center zone, rats that went through CUMS modeling presented depressive‐like behaviors such as anhedonia and low locomotor activities. Rats in all groups except for the CON group got significant weight loss and reduced sucrose preference after 21‐day CUMS stimulation, revealing the success of the model establishment. Nevertheless, sucrose preference in SSRI and SSRI + EA groups increased but not EA group. As for locomotor activities, the total distance moved was longer in EA and SSRI + EA groups than that in CUMS group, while time spent in the center zone was the longest in the SSRI group. Taking the evidence together, EA and SSRI + EA were effective in ameliorating rats' depressive‐like behaviors, which was similar to positive control medicine SSRI.

Based on our former research,^19^ we further explored the vmPFC in CUMS rats in this study, now that the PFC is noteworthy for the realization of mood, learning, memory, execution, information integration, and other functions. The vmPFC in PFC integrates information from other brain regions such as the amygdala, striatum, ventral tegmental area (VTA), and temporal lobe, leading to the generation of negative emotions, self‐awareness, and self‐reflection. vmPFC's importance has already been proven in the antianxiety and antiaddiction effect of EA,^47^, ^48^ which is a hint about EA's mechanism underneath the comorbidity of depression and anxiety or addiction.

In line with previous findings,^42^, ^49^ the present study observed inhibition of neuronal activities (reduced amplitude of spEPSC) in the vmPFC and the promotion of depressive‐like behaviors, which were reversed by EA. Medial PFC (mPFC) consists of excitatory pyramidal neurons, the majority, and inhibitory interneurons.^50^ The homeostasis of excitatory and inhibitory signaling has been observed in the mPFC of depression‐related behaviors.^43^, ^51^ Changes in spEPSC and spIPSC represent neuronal activities, which are closely associated with synaptic transmission. To phrase it, the downregulated amplitude of spEPSC in the vmPFC indicated the role defeated synaptic transmission plays in the pathogenesis of depression.

In this experiment, EA improved the depression of rats by enhancing spontaneous excitatory synaptic signaling in vmPFC. Although the influence of EA on EPSC in vmPFC is not clear currently, several studies have demonstrated that depressive‐like behaviors were associated with the excitability of layer 5 pyramidal neurons in the mPFC.^44^ Moreover, EA could reverse the synaptic spontaneous release^52^ and ameliorate sleep‐deprivation‐induced depression‐like behaviors by enhancing long‐term potentiation (LTP, a long‐lasting enhancement of synaptic transmission efficacy)^53^ in the hippocampus. The evidence may enlighten the mechanism of EA on synaptic spontaneous release in vmPFC of depression treatment.

From the results, the expression level of DAT rose in CUMS model rats' vmPFC. In fact, the changes in DAT were controversial in patients with depression, in which upregulated or diminished DAT expression was both observed.^54^, ^55^ On the whole, a majority of studies still support the high DAT expression level in depression, and PET research considered that decreased striatal DAT expression might be a compensatory downregulation due to low DA signaling within mesolimbic pathways.^56^ DA system is a major component in the development of depressive phenotypes related to stress.^46^ In addition to DAT and common D1‐like or D2‐like receptors which partake in regulating synaptic transmission, DA system also correlates with interneurons or astrocytes in several brain regions.^57^, ^58^ Abnormal neuron discharge and downregulation of the DA system have been linked to depressive‐like states.^46^ Located in the presynaptic membrane, DAT regulates the content of DA through the reuptake of DA into presynaptic cells, thus affecting the efficiency of neurotransmission. The inhibition of DAT increased synaptic plasticity in hippocampus and enhanced transsynaptic levels of DA in the PFC, which could reverse depressive‐like behaviors and restore motivational deficiencies appearing in depression.^45^

In this study, EA achieved an antidepressant effect with decreased total and phosphorylated DAT, as well as the reversal of DAT phosphorylation after CUMS. Taking together with the behavioral results, our study demonstrated that EA's effect on depression was related to the phosphorylation of DAT but not merely the expression of DAT or p‐DAT in vmPFC. Phosphorylation is one of the main post‐translational modifications of DAT, which could upregulate extracellular DA, increase locomotor activities, and present antidepressant effects.^59^, ^60^, ^61^ At present, the phosphorylation of DAT has been learned thoroughly, but it has been hardly discussed in depression. Our experiment initially observed the consistent changes of spontaneous excitatory synaptic signaling and DAT phosphorylation by EA, indicating an underlying mechanism of EA on depression improvement.

Here, ameliorated depression was along with raised TAAR1 and PKA expression of rats after EA treatment. TAAR1 is a novel target gathering more and more attention in regulating the dopaminergic nervous system and related disorders.^31^, ^62^ As an intracellular G protein‐coupled receptor (GPCR), TAAR1 elevates cAMP levels and downstream signal transduction such as PKA, which is also known as cAMP‐dependent PKA. From our results, EA increased PKA level but not cAMP in vmPFC of depression model rats. Such PKA activation of EA was consistent with research studying depression.^63^, ^64^ The phosphorylation by PKA on monoamine transporters can regulate the signaling cascades of corresponding neurons.^65^ This research showed that the phosphorylation of DAT was connected with cAMP and PKA activation, and the upstream activator GPCR may be TAAR1.

This article is the first to discuss the influence of EA on TAAR1 using CUMS model. We hypothesized that EA's effect on depression may be connected with TAAR1's regulation in the monoaminergic system. Nowadays, the antidepressant effect of TAAR1 has been recognized in many studies. TAAR1 can be detected intriguingly in both PFC and monoaminergic areas, such as the hippocampus, amygdala, substantia nigra, and VTA in rodent animals.^66^ A depression animal model with cognitive dysfunction (chronic social defeat stress) demonstrated the downregulated expression level of TAAR1 in the mPFC,^67^ and the activation of this receptor may be beneficial to depressive symptoms in other mental disorders such as schizophrenia by increasing neural activity and upregulates the expression of plasticity‐related genes in prefrontal areas.^30^, ^68^ EA has been confirmed to increase DA release in depressed animals.^11^ In TAAR1‐knockout mice, the selective TAAR1 agonist's effect in the forced swim test (a typical test measuring depression of rodents) was blunted and the extracellular DA in PFC was reduced.^69^ In neurons with co‐localization of TAAR1, TAAR1 agonists increase the concentration of related monoamines in the synaptic clefts, thereby increasing the binding of postsynaptic receptors.^70^ Besides, TAAR1 is also closely related to drug addiction like methamphetamine or cocaine overdose. EA can help to improve psychiatric symptoms, anxiety, and depression in methamphetamine addicts during abstinence, and promote rehabilitation of patients.^71^ These findings can provide enlightenment on EA's molecular mechanism of action in depression by regulating TAAR1.

As for electrophysiology, research also showed that TAAR1 activation reversed the impaired excitatory/inhibitory (E/I) balance in the mPFC of depression model animals, which is maintained by glutamatergic excitatory synapses and GABAergic inhibitory synapses.^67^ Although the E/I ratio wasn't calculated in this experiment, counting on the increased spEPSC and nonsignificant changes of spIPSC, EA tended to present a similar E/I variation trend as TAAR1 in depression treatment.

Some limits still existed in our experiment. First of all, EA's effect on improving sucrose preference was less potent than that of SSRI. Second, we did not measure extracellular dopamine levels or record LTP in vmPFC. Third, the electrophysiological mechanism under SSRI or SSRI + EA to depression has not been studied. In the future, activating or inhibiting TAAR1 will be applied to further reveal the mechanism of this receptor in regulating depression.

## 5 CONCLUSION

Accumulating results, the antidepressant effect of EA was associated with enhanced synaptic transmission in vmPFC. Biomolecularly, EA increased the declined phosphorylation of DAT and TAAR1 expression in CUMS rats, which may be relevant to cAMP and PKA. Therefore, DAT phosphorylation participated in the promotion of synaptic transmission in vmPFC by EA with TAAR1, cAMP, and PKA involved.

## AUTHOR CONTRIBUTIONS

Yong Huang and Siqiang Ren designed and supervised the study. Xiaowen Cai performed experiment procedures, analyzed data, and undertook the statistical analysis. Xiaowen Cai and Mei Wu drafted the manuscript. Huacong Liu, Shengtao Huang, and Jia Song participated the experiment procedures. The manuscript with revisions was approved by all authors.

## FUNDING INFORMATION

This work was supported by the National Natural Science Foundation of China (81873359 and 82074519); the Natural Science Foundation of Guangdong Province (2022A1515011658 and 2022A1515010219); Sanming Project of Medicine in Shenzhen (SZZYSM202108013); and the Student's Platform for Innovation and Entrepreneurship Training Program of Southern Medical University of China (No. 202212121063).

## CONFLICT OF INTEREST STATEMENT

The authors declare that there is no conflict of interest related to the study.

## Supporting information


Data S1.

## Data Availability

The data that support the findings of this study are available from the corresponding author upon reasonable request.
