# Supplementary material for: Electroacupuncture alleviated depression‐like behaviors in ventromedial prefrontal cortex of chronic unpredictable mild stress‐induced rats: Increasing synaptic transmission and phosphorylating dopamine transporter
Source: CNS Neurosci Ther. 2023 Apr 1;29(9):2608–20. doi: 10.1111/cns.14200 (PMC10401110; doi:10.1111/cns.14200)

Supplementary information for

Electroacupuncture alleviated depression-like behaviors in ventromedial prefrontal cortex of chronic unpredictable mild stress-induced rats: Increasing synaptic transmission and phosphorylating dopamine transporter

Xiaowen Cai, Mei Wu, Zhinan Zhang, Huacong Liu, Shengtao Huang, Jia Song, Siqiang Ren, Yong Huang

Xiaowen Cai is the first author and Mei Wu is the co-first author. Xiaowen Cai and Mei Wu contributed equally in this manuscript.
Correspondence should be addressed to Yong Huang and Siqiang Ren.

This file includes:
Original gel/blot images of each cropped gel/blot appearing in the manuscript.

Full unedited gel/blots for Figure 5

Dopamine transporter (DAT)

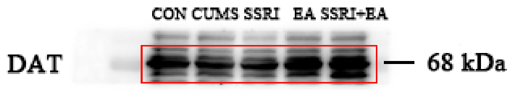

Phosphorylated DAT (p-DAT)

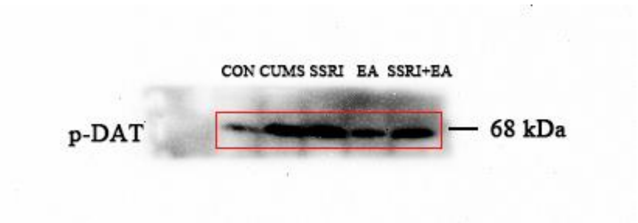

GAPDH

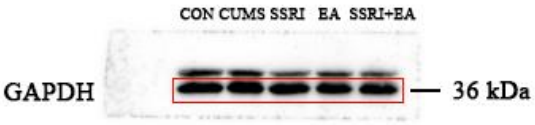

Full unedited gel/blots for Figure 6

Protein kinase A (PKA)

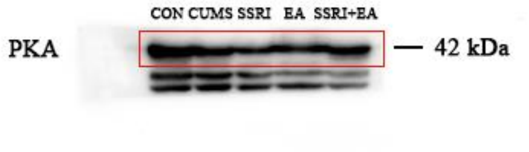

Trace amine-associated receptor 1 (TAAR1)

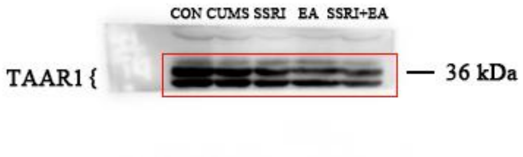

GAPDH

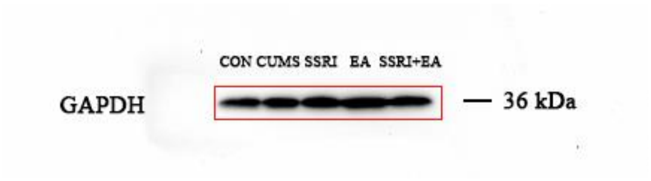

Supplement: Supplementary file 1 — Data S1. [file CNS-29-2608-s001.pdf]
